# Psychoses of the War, Including Neurasthenia and Shell Shock

**Published:** 1919-12

**Authors:** 


**Psychoses of the War, including Neurasthenia and Shell Shock.** By H. C. MARR, M.D. Pp. xiii., 292. London: Oxford Medical Publications. 1919. Price 16s. net.

Dr. Marr gives in this book a full and vivid account of the different types of psychoses which he has met with during and as the result of the war. He has had wide experience of such cases in the later stages both in Malta and as Neurological Consultant in the Scottish Command.

The clinical descriptions are excellent, although occasionally one is inclined to recall C. S. Myers' warning: "In this country, at least, we have been paying so much attention to the mental aspect of the war neuroses that a detailed examination of the accompanying bodily symptoms has been generally neglected." Dr. Marr finds room to include as psychoses of the war a considerable group of physical injuries and diseases under his classification of organic neurasthenia. Your reviewer, with experience of and wide responsibility for cases of war neuroses in front line units, found that it was of the utmost importance to weed out the organic injuries and diseases, especially the acute and chronic infections, from the purely psychical cases at the earliest possible opportunity.

It is striking to find many cases reported without reference to the question of previous syphilitic infection, and apparently without resort to the Wassermann reaction.

Still, as a study of war psychoses from the mental view point it is an interesting contribution. The methods of observation and case taking in the appendix which follows the general index are helpful and suggestive. There full attention is directed to the "examination of the general bodily system."

On the whole this is not a book for beginners in the subject. The classification of neurasthenia, and the views on cerebral localisation of psychical processes are not those universally accepted. But it will be read with profit and pleasure by those who with some knowledge of other views on psychology and with first-hand experience of war neuroses can appreciate the undoubted value of Dr. Marr's observations.

The volume is got up in the excellent manner we have grown to associate with the Oxford Medical Publications, and it is of handy size.

